# Inflammatory profiles in canine intervertebral disc degeneration

**DOI:** 10.1186/s12917-016-0635-6

**Published:** 2016-01-13

**Authors:** Nicole Willems, Anna R. Tellegen, Niklas Bergknut, Laura B. Creemers, Jeannette Wolfswinkel, Christian Freudigmann, Karin Benz, Guy C. M. Grinwis, Marianna A. Tryfonidou, Björn P. Meij

**Affiliations:** Department of Clinical Sciences of Companion Animals, Faculty of Veterinary Medicine, 3584 CM Utrecht, The Netherlands; Department of Orthopaedics, University Medical Center, 3584 CX Utrecht, The Netherlands; NMI Natural and Medical Sciences Institute at the University of Tuebingen, Regenerative Medicine II, 72770 Reutlingen, Germany; Department of Pathobiology, Faculty of Veterinary Medicine, 3508 TD Utrecht, The Netherlands

**Keywords:** Low back pain, Dog, PGE_2_, Prostaglandin, Herniation, Chondrodystrophic, Non-chondrodystrophic

## Abstract

**Background:**

Intervertebral disc (IVD) disease is a common spinal disorder in dogs and degeneration and inflammation are significant components of the pathological cascade. Only limited studies have studied the cytokine and chemokine profiles in IVD degeneration in dogs, and mainly focused on gene expression. A better understanding is needed in order to develop biological therapies that address both pain and degeneration in IVD disease. Therefore, in this study, we determined the levels of prostaglandin E2 (PGE_2_), cytokines, chemokines, and matrix components in IVDs from chondrodystrophic (CD) and non-chondrodystrophic (NCD) dogs with and without clinical signs of IVD disease, and correlated these to degeneration grade (according to Pfirrmann), or herniation type (according to Hansen). In addition, we investigated cyclooxygenase 2 (COX-2) expression and signs of inflammation in histological IVD samples of CD and NCD dogs.

**Results:**

PGE_2_ levels were significantly higher in the nucleus pulposus (NP) of degenerated IVDs compared with non-degenerated IVDs, and in herniated IVDs from NCD dogs compared with non-herniated IVDs of NCD dogs. COX-2 expression in the NP and annulus fibrosus (AF), and proliferation of fibroblasts and numbers of macrophages in the AF significantly increased with increased degeneration grade. GAG content did not significantly change with degeneration grade or herniation type. Cytokines interleukin (IL)-2, IL-6, IL-7, IL-8, IL-10, IL-15, IL-18, immune protein (IP)-10, tumor necrosis factor (TNF)-α, and granulocyte macrophage colony-stimulating factor (GM-CSF) were not detectable in the samples. Chemokine (C-C) motif ligand (CCL)2 levels in the NP from extruded samples were significantly higher compared with the AF of these samples and the NP from protrusion samples.

**Conclusions:**

PGE_2_ levels and CCL2 levels in degenerated and herniated IVDs were significantly higher compared with non-degenerated and non-herniated IVDs. COX-2 expression in the NP and AF and reactive changes in the AF increased with advancing degeneration stages. Although macrophages invaded the AF as degeneration progressed, the production of inflammatory mediators seemed most pronounced in degenerated NP tissue. Future studies are needed to investigate if inhibition of PGE_2_ levels in degenerated IVDs provides effective analgesia and exerts a protective role in the process of IVD degeneration and the development of IVD disease.

**Electronic supplementary material:**

The online version of this article (doi:10.1186/s12917-016-0635-6) contains supplementary material, which is available to authorized users.

## Background

Intervertebral disc (IVD) disease is a common spinal disorder in dogs and humans and is characterized by clinical signs ranging from back pain to neurological deficits. IVD disease is preceded by IVD degeneration with a similar etiopathogenesis in dogs and humans [1]. Chondrodystrophic (CD) dogs are predisposed to explosive extrusion of the nucleus pulposus (NP) (Hansen type I) of degenerated thoracolumbar and cervical IVDs, mainly between 3 and 7 years of age. Non-chondrodystrophic (NCD) dogs are predisposed to protrusion of the annulus fibrosus (AF) (Hansen type II) of degenerated lumbosacral and caudal cervical IVDs at 6 to 8 years of age, and NP extrusion of degenerated thoracolumbar IVDs [2–5]. Hansen type II annular protrusion does occur in CD dogs, but less commonly [6, 7].

The onset of IVD degeneration at a cellular level is characterized by a gradual replacement of notochordal cells by chondrocyte-like cells in the NP. In this respect, the NP of CD dogs contains primarily chondrocyte-like cells already by one year of age, while notochordal cells remain the predominant cell type in the NP of NCD dogs during their lifetime. In the latter, notochordal cells in some IVDs are substituted and degeneration occurs at a much later age [1, 8, 9]. In both types of dogs, a decrease in proteoglycan content, a shift in collagen type II to collagen type I in the extracellular matrix of the NP, together with a disruption of the lamellae in the annulus fibrosus (AF) is seen during IVD degeneration [10, 11]. Furthermore, in degenerated discs, nerve endings extend into the deeper layers of the AF and into the NP, in contrast to healthy discs, in which only the outer third of the AF is innervated. Stimulation of nociceptors in the AF and dorsal longitudinal ligament is related to pain [12, 13]. A nociceptive response can either be evoked by a mechanical or inflammatory stimulus. Various inflammatory mediators have also been suggested to play a role in the catabolic processes in human NP and AF tissue, including prostaglandin E_2_ (PGE_2_), interleukins (IL-1α, IL-1β IL-6, IL-8), and tumor necrosis factor α (TNF-α) [14–16]. In NP cells from experimental CD dogs with surgically induced IVD degeneration increased levels of TNF-α and IL-1β were shown in vitro [17]. While knowledge of the involvement of inflammatory mediators in human IVD degeneration has substantially increased over the last years [14–16, 18–34], only limited studies have focused on cytokine and chemokine profiles in IVD degeneration in dogs, and mainly focused on gene expression [17, 35, 36].

PGE_2_ is the most common prostanoid and plays an important regulatory role in physiological as well as pathological processes. It is synthesized by two cyclooxygenase (COX) isoforms, COX-1 and COX-2, by conversion of arachidonic acid into prostaglandin H_2_ (PGH_2_) and isomerization of PGH_2_ to PGE_2_ by prostaglandin E synthases. COX-2 expression is highly restricted under physiological conditions, but can be rapidly induced in response to inflammatory stimuli and is therefore believed to play an important role in the PGE_2_ production involved in degenerative processes [27, 37]. Current therapies of IVD disease aim at alleviating pain by administration of corticosteroids, non-steroidal anti-inflammatory drugs (NSAIDs), and/or opioids, by physical therapy, or by surgery. Among the numerous NSAIDs available, oral selective COX-2 inhibitors are primarily used for managing clinical signs, as they reduce inflammation and relieve pain, but cause less gastrointestinal side effects [38, 39]. Delivery of COX-2 inhibitors directly into the avascular IVD has been suggested as an alternative route of administration to enhance the local efficacy and to minimalize systemic side effects. A recent study in experimental CD dogs has shown the biocompatibility and safety of intradiscal injection of a hydrogel loaded with a selective COX-2 inhibitor [40]. As PGE_2_ is one of the inflammatory mediators in human IVD herniation that has been shown to sensitize nerves and induce pain, the efficacy of intradiscal delivery of NSAIDs is likely to be limited to IVD disease with a clear inflammatory profile [41]. We hypothesize that PGE_2_ levels are higher in degenerated and herniated (protruded or extruded) IVDs of CD and NCD dogs compared with non-degenerated or non-herniated IVDs. Therefore, we determined the levels of PGE_2_, cytokines, chemokines, and matrix components in IVDs from CD and NCD dogs with and without clinical signs of IVD disease and correlated these to degeneration grade or herniation type. In addition, we investigated COX-2 expression in histological IVD samples of CD and NCD dogs.

## Methods

### Collection and preparation of samples for biochemical analyses

#### IVDs collected post-mortem

A total of 19 IVDs, with a Thompson grade I and II, were collected from 7 laboratory (3 CD, 4 NCD) dogs that were euthanized in unrelated animal experiments (experiment numbers: DEC 2007.III.08.110, DEC 2009.III.06.050),and a total of 34 samples, with a Pfirrmann grade II, were collected from 15 laboratory (CD) dogs from previous animal experiments (DEC 2012.III.05.046, DEC 2013.III.02.017). All animal experiments were approved by the Ethics Committee of Animal Experiments (DEC) of Utrecht University. None of the dogs had a history of clinical signs of IVD disease. NP and AF tissues were isolated from the spine and collected separately, snap frozen in liquid nitrogen, and stored at −80 °C until further analysis.

#### IVDs collected during surgical treatment

A total of 123 IVDs were collected from 76 client-owned dogs that were referred to the University Clinic for Companion Animals in Utrecht with IVD disease that required surgical intervention. The diagnosis of IVD disease was confirmed on MRI or CT. Dogs were classified as CD and NCD and divided into subgroups, based on the Pfirrmann grade on T2-weighted MR images [42, 43]. In 10 samples of 6 dogs, in which no MRI was available, grading of the IVD was performed on CT images [43]. The medical history and records of the dogs were screened for information on prior medical treatment of IVD disease and the duration of this treatment.

#### Diagnostic imaging

Diagnostic imaging was performed in fully anesthetized client-owned dogs, according to standard practice. MRI images were obtained with a 0.2 Tesla open MRI system (Magnetom Open Viva, Siemens AG, Erlangen, Germany) by using multipurpose flex coils until 2013, and thereafter with a 1.5 Tesla scanner (Ingenia, Philips Healthcare, Best, The Netherlands) by using a small-extremity or a posterior coil. For each examination a coil was chosen that fitted around the body of the patient as closely as possible. Sagittal T2-weighted (T2W) images were acquired using a turbo-spin echo pulse sequence with the following parameters: repetition time = 2500–3048 ms, echo time = 110–120 ms, field of view = 50 x 160/160 x 350 mm, acquisition matrix = 100 x 256/200 x 235 mm, voxel size = 0.6 x 0.8/0.8 x 1.03 mm slice thickness = 2 – 2.5 mm. CT images were obtained with a third-generation single-slice helical CT-scanner (Philips Secura). Contiguous 2 mm thick slices with 1 mm overlap were obtained with exposure settings of 120 kV and 260 mA.

#### Surgical treatment

Client-owned dogs were anesthetized according to standard of care. Collection of IVDs was achieved through standard surgical procedures, depending on the location of disc herniation: ventral decompression in the cervical area, dorsolateral hemilaminectomy in the thoracolumbar area, and dorsal laminectomy in the lumbosacral area. In dogs with nuclear extrusion (Hansen type I), free NP material was collected from the epidural space in the spinal canal, and AF material was collected during ventral fenestration preceding the ventral decompression for cervical disc herniations, or when an additional lateral fenestration was performed after hemilaminectomy for thoracolumbar disc herniations. In dogs with lumbosacral annular protrusion (Hansen type II), partial discectomy consisting of annulotomy and nucleotomy, allowed separate collection of NP and/or AF tissue. In 3 dogs, an adjacent IVD was fenestrated, and AF and/or NP material was collected. The treatment decision (discectomy, nucleotomy, fenestration) was taken during surgery and depended on the state of the AF and the position of the NP. Each surgeon documented the type of herniation (NP extrusion (Hansen type I) or AF protrusion (Hansen type II)) and type of collected material (NP or AF) in the surgical report.

NP and/or AF tissues were collected in separate vials during surgery, snap frozen into liquid nitrogen within minutes after collection, and subsequently stored at −80 °C until further analysis. Details of the samples are shown in Table 1 and in Additional file 1.

**Table 1:** Sample classification details

Samples biochemistry					Samples histopathology		
# Dogs	CD dogs		NCD dogs		# Dogs	CD dogs	NCD dogs
	58		40			15	10
Age – median	5 years		2 years		Age – median (range(yrs))	10 year	7 years
(range(mths – yrs))	(1–11 )		(8–12)			(2–10)	(1–10)
Spinal location	NP	AF	NP	AF	Spinal location		
# IVDs	35	30	36	41	# IVDs	19	18
Cervical (C1 – T1)	13	21	10	16	Cervical (C1 – T1)	ND	ND
Thoracolumbar (T1 – L1)	21	12	2	ND	Thoracolumbar (T1 – L1)	5	8
Lumbar (L1 – S1)	11	7	23	25	Lumbar (L1 – S1)	14	10
Unknown	7	7	1	ND	Unknown	ND	ND
Degeneration					Degeneration		
Grade 1 (non-surgical)	ND	ND	9^a^	10^a^	Grade 1	2	8
Grade 2	32^b^	32^c^	9	9	Grade 2	4	7
Grade 3	7	6	8	8	Grade 3	3	3
Grade 4 + 5	13^d^	9^d^	10	12 + 2	Grade 4 + 5	3 + 3	3
Displacement					Displacement		
NP in situ (non-surgical)	27^b,c^	27^b,c^	9^a^	10^a^	NP in situ	ND	
Nuclear extrusion	24	18	12	7	Nuclear extrusion	ND	
Annular protrusion	1	2	15	24	Annular protrusion	ND	
Treatment					Treatment		
No treatment	9	9	14	20	No treatment	ND	
Treatment	24	21	20	20			
NSAID < 1 wk	7	8	7	4	NSAID < 1 wk	ND	
NSAID > 1 wk	6	7	8	9	NSAID > 1 wk	ND	
Steroids < 1 wk	4	2	1	ND	Steroids < 1 wk	ND	
Steroids > 1 wk	4	3	2	2	Steroids > 1 wk	ND	
Other medication	3	1	2	5	Other medication	ND	
Unknown	2	ND	2	1	Unknown	ND	

^a^Samples collected from experimental dogs

^b^9 samples collected from experimental dogs (non-surgical); 17 samples collected in a previous study [40]

^c^4 samples collected from experimental dogs (non-surgical); 17 samples collected in a previous study [40]

^d^1 sample collected via fenestration

^e^Samples collected from experimental dogs;17 samples collected in a previous study [40]

#### Biochemical analyses of IVDs collected post-mortem and intra-operatively

Prior to analyses, samples were weighed, and 400 μl and 750 μl lysis buffer (cOmplete lysis M EDTA buffer, Roche diagnostics Nederland B.V., Almere, The Netherlands) was added to NP and AF tissue, respectively. Tissues were lysed in a TissueLyser II (Qiagen, Venlo, The Netherlands) for 2x 60 s at 20 kHz. After centrifugation for 15 min at 14.000 g, the volume of the supernatant of each sample was measured and separated from its pellet. A volume of 80 μl was filtered over a 0.22 μm nylon spin-X centrifuge tube filter (8169, Costar, Corning Incorporated, NY, USA) and stored at −80 °C in aliquots for cytokine measurements.

##### Glycosaminoglycan and DNA assays

To determine GAG and DNA, supernatants and pellets were digested in a papain buffer (250 μg/ml papain (P3125-100 mg, Sigma-Aldrich) + 1.57 mg cysteine HCL (C7880, Sigma-Aldrich)) at 60 °C overnight. The 1.9-dimethylmethylene blue (DMMB) assay was used to determine GAG content [44]. A volume of 16 mg DMMB (341088 Sigma-Aldrich) was added to 5 ml 100 % ethanol and incubated overnight on a roller bench. A solution of 2.37 g NaCl and 3.04 g glycine in 1 l distilled water with a pH set at 3.00 was sterilized by using a 0.22 μm syringe filter (SLGSV255F Millex-GS Syringe Filter Unit, Merck Millipore, Darmstadt, Germany), added to the DMMB solution, and stored at 4 °C, protected from light. Pellets were diluted 1:1000 and supernatants 1:150 in PBS-EDTA. A volume of 100 μl of the dilutions and standards was pipetted into a 96-wells microplate (655199 PS microplate, Greiner Bio-One, Alphen aan den Rijn, Netherlands), and prior to spectrophotometric analysis, 200 μl of DMMB was added to each well. The ratio of absorption at 540 to 595 nm was measured by using a microplate reader (Multimode detector DTX 880, Beckman Coulter). Chondroitin sulphate from shark cartilage (C4384, Sigma-Aldrich) was used as a standard to calculate GAG concentrations. The Quant-iT™ dsDNA Broad-Range assay kit in combination with a Qubit Fluorometer (Invitrogen, Carlsbad, USA) was used according to the manufacturer’s protocol to determine the DNA content.

##### PGE_2_, and cytokine assays

PGE_2_ levels were determined in the supernatants by using a colorimetric competitive enzyme immunoassay kit (PGE_2_ high sensitivity EIA kit, ENZO Life Sciences BVBA, Antwerp, Belgium). A magnetic canine cytokine bead panel based on Luminex® xMAP® technology (#CCYTOMAG-90 K/CCYTOMAG-90 K-PX13); Milliplex® MAP kit, Millipore Corporation, Billerica, USA) was used to measure twelve different cytokines and chemokines in supernatants: TNF-α, granulocyte-macrophage colony-stimulating factor (GM-CSF), IL-2, IL-6, IL-7, IL-8, IL-10, IL-15, IL-18, chemokine (C-C motif) ligand 2 (CCL2), chemokine (C-X-C motif) ligand 1 (CXCL1), chemokine (C-X-C motif) ligand 10 (CXCL10). Supernatants were diluted 1:2 and were measured according to the manufacturer’s instructions. All biochemical values were corrected for weight of the sample.

### Collection of post-mortem IVDs for histology

#### IVDs collected post-mortem

Post-mortem, 37 IVDs were collected from vertebral columns of 16 client-owned dogs that were euthanized for diseases other than IVD disease and submitted for necropsy to the Department of Pathobiology at the Faculty of Veterinary Medicine, Utrecht University, and from 9 experimental dogs in unrelated cardiovascular experiments (DEC 2007.II.01.029, DEC 2011.07.065). Permission to collect material from the client-owned dogs was granted by the owners. None of the dogs had a reported history of back problems. Details of the dogs of which material was collected for histology are shown in Table 1.

#### Diagnostic imaging

Within 24 h after euthanasia or death, the vertebral column (T11 – S1) was harvested by using an electric multipurpose saw (Bosch, Stuttgart, Germany). Within 1 h after dissection, sagittal T2W MR images were obtained with a 0.2 Tesla open MRI system (Magnetom Open Viva, Siemens AG) as described earlier. All lumbar IVDs were graded on midsagittal T2W images according to the Pfirrmann score by two independent investigators (NW, AT) [42].

#### Histology and immunohistochemistry

After scanning, all muscles were removed and the vertebrae were transected transversely with a band saw (EXAKT tape saw, EXAKT Advanced Technologies GmbH, Norderstedt, Germany), resulting in spinal units (endplate – IVD – endplate). These units were then transected sagittally into two halves by using a diamond band pathology saw (EXAKT 312 saw; EXAKT diamond cutting band 0.1 mm D64; EXAKT Advanced Technologies GmbH, Norderstedt, Germany). Midsagittal slices (3 – 4 mm) were cut from one half and fixed in 4 % neutral buffered formaldehyde and decalcified in EDTA. Samples were dehydrated in graded alcohol series, rinsed in xylene, and embedded in paraffin. Sections (5 μm) were cut, deparaffinized and rehydrated, and stained with both hematoxylin (109249, Merck)/eosin (115935, Merck), and with picrosirius red (saturated aqueous picric acid: 36011, Sigma-Aldrich, sirius red: 8015, Klinipath)/alcian blue (alcian blue: 05500, Sigma-Aldrich; glacial acetic acid: 100063, Merck). Histological sections were assessed for the presence of inflammatory cells, and evaluated according to a histological grading scheme described by Bergknut et al. [45].

Immunohistochemistry for COX-2 was performed on 5 μm sections mounted on KP plus glass slides (Klinipath B.V., Duiven, The Netherlands). After deparaffinization and rehydration sections were treated with Dual Endogenous Enzyme Block (S2003, Dako, California, USA) for 10 min at room temperature to block nonspecific endogenous peroxidase, followed by 2 washing steps of each 5 min with tris buffered saline containing 1 % Tween 20® (TBS-T). Sections were treated with TBS bovine serum albumin (BSA) 5 % solution to block non-specific binding for 60 min at room temperature. Subsequently they were incubated with a primary mouse anti-human monoclonal COX-2 antibody (#160112 Clone CX229, Cayman, Ann Arbor, USA) diluted 1:800 in TBS-BSA 5 % overnight at 4 °C. The following day sections were incubated with peroxidase-labelled polymer (K4007; Envision anti-mouse, Dako) and antibody binding was visualized by using diaminobenzidine (DAB; K4007; Dako). Sections were counterstained with hematoxylin solution (Hematoxylin QS, Vector, Peterborough, UK), rehydrated and mounted in permanent mounting medium. The percentage of COX-2 positive chondrocytes in the NP, and in the dorsal AF (DAF) was determined by manual counting by a blinded independent investigator (AT).

#### Statistical analyses

Data were analyzed by using R statistical software, package 2.15.2 (http://www.r-project.org/). A multiple linear regression model was used to analyze the effect of multiple explanatory variables on corrected PGE_2_, GAG, and DNA levels for the wet weight of the tissues. Furthermore, in order to be able to compare this study with previous reports [40], PGE_2_ levels were also corrected for DNA content. Data were logarithmically transformed to achieve normality. Two separate models were employed to investigate the association of explanatory variables ‘grade’ (Pfirrmann grade I – IV) and ‘herniation’ (NP in situ, NP extrusion, AF protrusion) with inflammatory parameters. Variables incorporated into both models were ‘dog’ (CD, NCD), ‘tissue’ (NP and AF), ‘treatment’ (no treatment, NSAID administered less than (<) 1 wk, NSAID administered more than (>) 1 wk, corticosteroids (cort) < 1 wk, cort > 1 wk, other) and their interaction. Residual plots and quantile-quantile (QQ)-plots were used to check the critical assumptions of linearity, equal variance at all fitted values and the assumption of normally distributed residuals. The Cox proportional hazards regression model was used for analysis of the COX-2 values, that did not approximate a normal distribution after log transformation. ‘Grade’ (Pfirrmann grade I – IV) and ‘breed’ (CD, NCD) and their interaction were incorporated into this model. Calculations were performed on values distracted from 100 %. In the absence of COX-2 positive cells the sample was set at 100 % and right censored. Histological reactive changes in the IVDs were statistically evaluated by using the nonparametric Kruskal-Wallis test, followed by a Mann–Whitney U-test. The Spearman’s correlation coefficient was calculated to estimate the correlation between the presence of inflammatory cells (‘yes’ or ‘no’) and COX-2 positive cells.

For all statistical models, regression coefficients were estimated by the maximum likelihood method. Model selection was based on the lowest Akaike Information Criterion (AIC). Confidence intervals were calculated and stated at the 99 % confidence level to correct for multiple comparisons. Differences between treatments were considered significant if the confidence interval did not include 0, whereas hazard ratios were considered significant if the confidence interval did not include 1. Significant differences and the corresponding confidence intervals are represented in Additional file 2.

## Results

### Extracellular matrix components and inflammatory profiles in relation to stage of degeneration

**Fig. 1:**
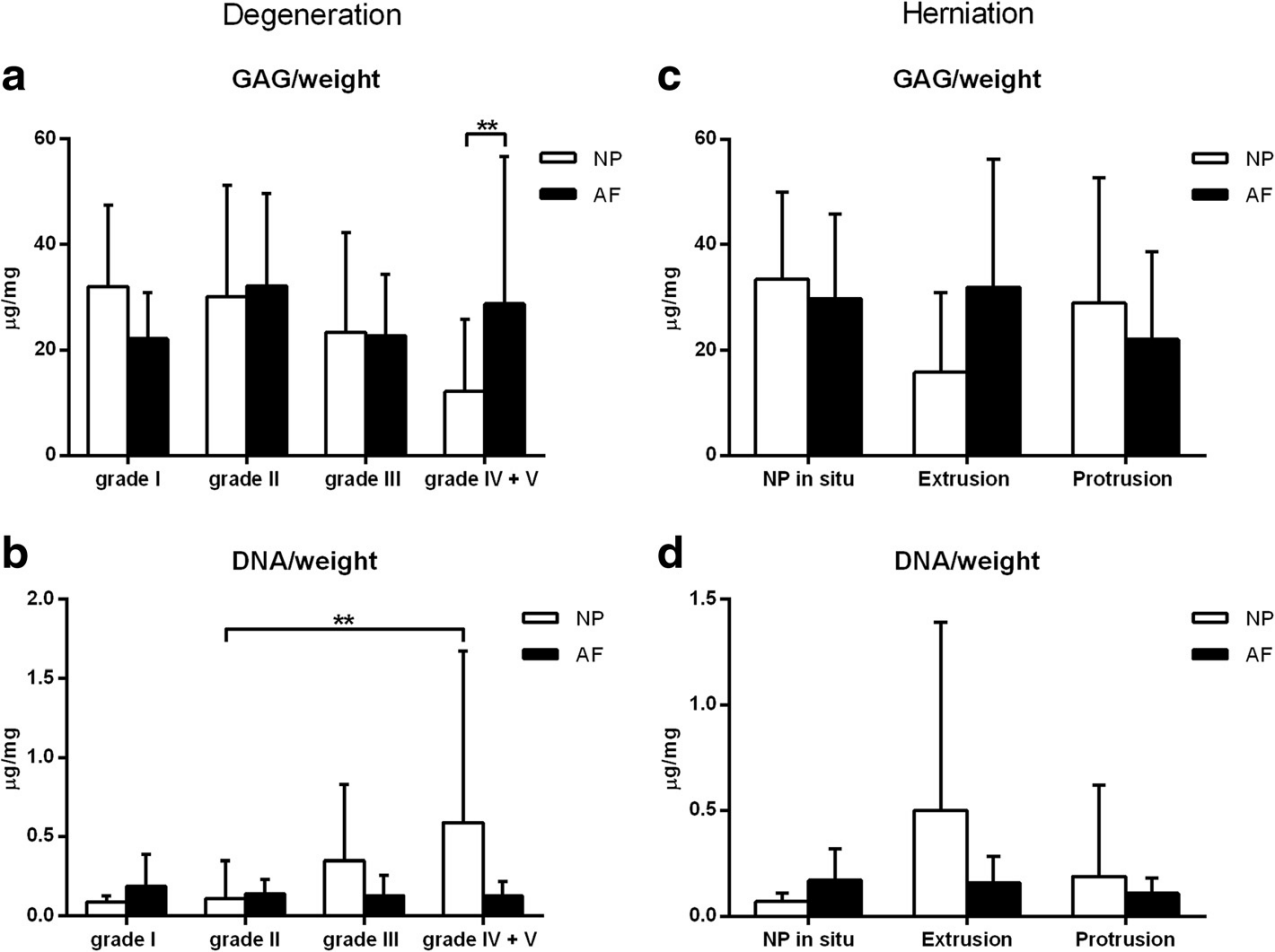
Mean + standard deviation GAG and DNA content normalized for weight in the nucleus pulposus (NP) and annulus fibrosus (AF) per Pfirrmann grade (**a**, **b**) and per herniation (**c**, **d**). **a**. GAG/weight levels were significantly higher in the AF compared with the NP in grade IV + V samples. **b**. DNA/weight was significantly lower in the NP of grade II samples compared with grade IV + V. **c** and **d**. Normalized GAG and DNA levels did not significantly differ between herniation groups. No significant differences were shown between dog (CD/NCD) or treatment (no treatment, NSAID < 1 wk, NSAID > 1 wk, corticosteroids (cort) < 1 wk, cort > 1 wk, other) groups, hence these groups are not shown separately. ** Indicate significant difference at a 99 % confidence level

**Fig. 2:**
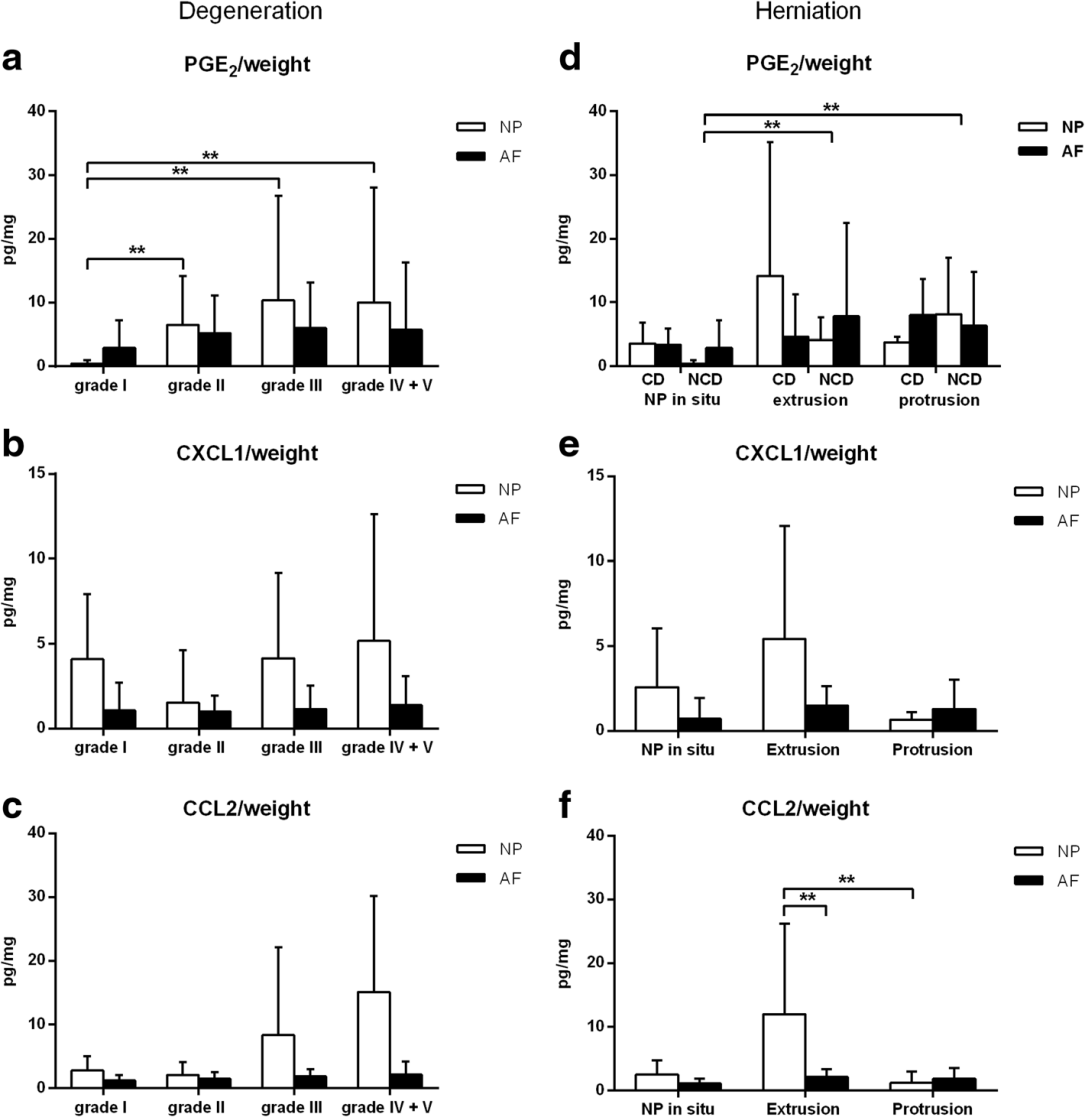
Mean + standard deviation PGE_2_ and chemokine (CCL2 and CXCL1) levels normalized for weight in the nucleus pulposus (NP) and annulus fibrosus (AF) per Pfirrmann grade (**a**, **b**, **c**) and per herniation (**d**, **e**, **f**). **a**. PGE_2_ levels expressed as pg/mg wet weight in grade I NP samples were significantly lower compared with grade II, III, and IV + V NP samples. **c**, **b** and **c**. CCL2 and CXCL1 levels normalized for weight did not significantly change with degeneration. **d**. PGE_2_ levels did not significantly differ in the NP and AF between the three herniation groups. **e**. CXCL1 levels did not significantly differ between herniation groups. **f**. CCL2 levels normalized for weight in the NP from extruded samples were significantly higher compared with the AF of these samples and the NP from protruded samples. No significant differences were shown between treatment (no treatment, NSAID < 1 wk, NSAID > 1 wk, corticosteroids (cort) < 1 wk, cort > 1 wk, other) groups, hence these groups are not shown separately. ** Indicate significant difference at a 99 % confidence level

GAG content normalized for wet weight did not significantly change with degeneration grade according to Pfirrmann (Fig. 1a and b). In grade IV + V samples the GAG content in the NP was significantly lower than in the AF (Fig. 1a). DNA expressed as μg/mg wet weight was significantly lower in grade II samples compared with grade IV + V samples (Fig. 1b). Due to sample limitations, samples that were above the upper range of the PGE_2_ assay (>1000 pg/ml) were set at 1000 pg/ml. PGE_2_ levels normalized for wet weight were significantly lower in grade I NP samples compared with those in grade II, III, and IV + V NP samples (Fig. 2a). PGE_2_ levels normalized for DNA were significantly lower in grade I samples compared with grade II, III, and IV + V samples regardless of the tissue origin (NP/AF), dog type (CD/NCD), or treatment group (no treatment, NSAID < 1 wk, NSAID > 1 wk, cort < 1 wk, cort > 1 wk, other) (Fig. 2b). Cytokines IL-2, IL-6, IL-7, IL-8, IL-10, IL-15, IL-18, IP-10, TNF-α, and GM-CSF were not detectable in the samples. Chemokine CCL2 was measured in 66/120 samples and chemokine CXCL1 in 119/120 samples. CXCL1 and CCL2 expressed as pg/gram wet weight did not significantly change with degeneration (Fig. 2c and d). There were no significant differences between treatment groups.

### Extracellular matrix components and inflammatory profiles in relation to herniation of NP and AF

GAG and DNA (Fig. 1c and d) and CXCL1 (Fig. 2d and e) expressed as pg/gram wet weight were not significantly different between herniation groups. PGE_2_ levels normalized for either DNA content or wet weight, were significantly lower in non-herniated samples compared with extruded and protruded samples of NCD dogs, regardless of the tissue origin (NP/AF), or treatment group (no treatment, NSAID < 1 wk, NSAID > 1 wk, cort < 1 wk, cort > 1 wk, other). CCL2 levels in the NP from extruded samples were significantly higher compared with the AF of these samples and the NP from protrusion samples regardless of the dog breed (CD/NCD) (Fig. 2f). There were no significant differences between biochemical parameters of CD and NCD dogs or treatment groups.

**Fig. 3:**
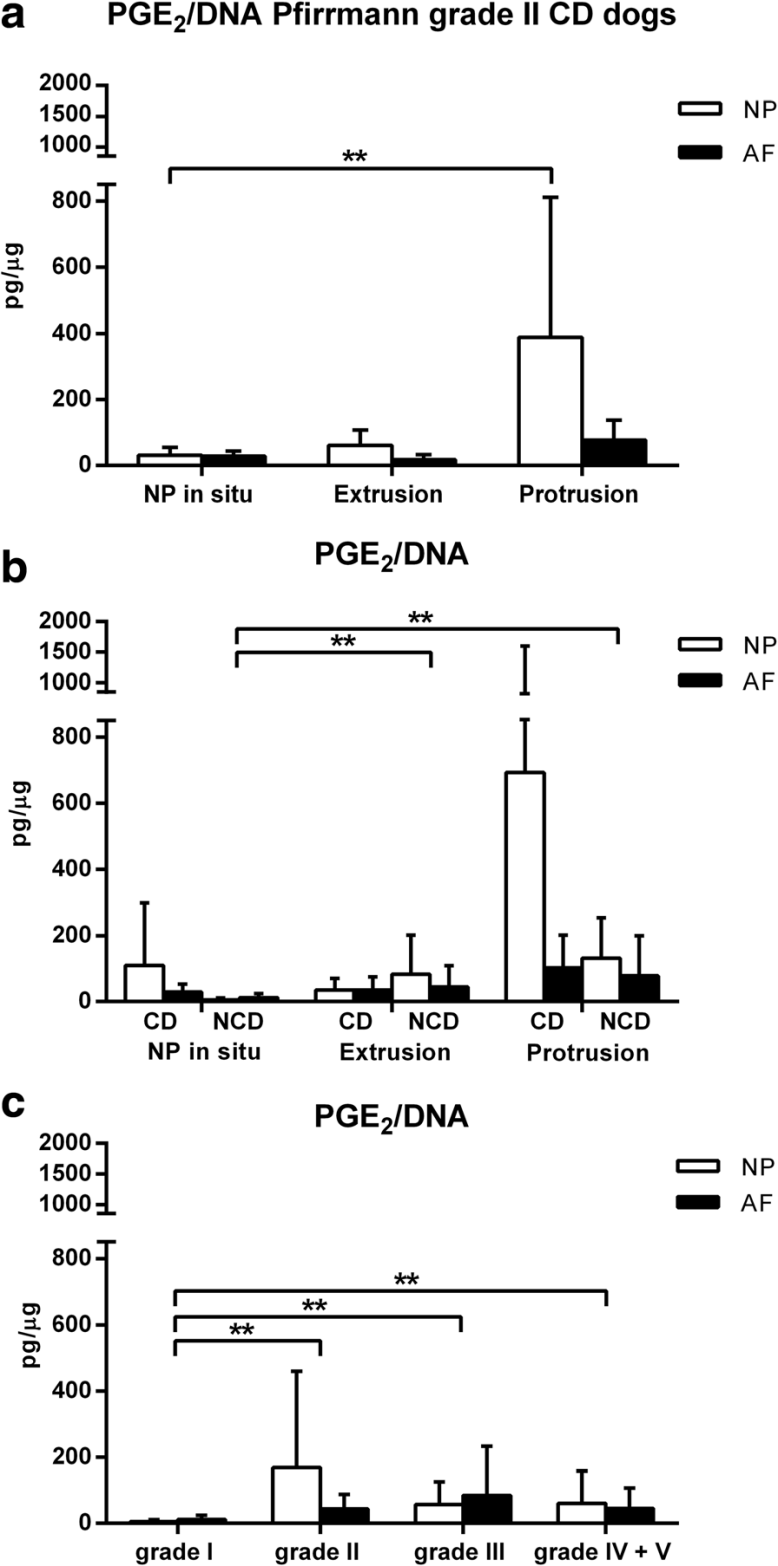
Mean + standard deviation PGE_2_ levels normalized for DNA content in the nucleus pulposus (NP) and annulus fibrosus (AF) in Pfirrmann grade II samples obtained from experimental chondrodystrophic (CD) dogs (**a**), and in the complete dataset (CD and nonchondrodystrophic (NCD) dogs) per Pfirrmann grade (**b**), and per herniation (**c**). **a**. PGE_2_ levels expressed as pg/μg DNA in the NP of CD dogs were significantly higher in extruded grade II samples compared with NP in situ samples. **b**. PGE_2_ levels normalized for DNA were significantly lower in grade I samples compared with grade II, III, and IV + V samples regardless of the tissue origin (NP/AF), dog group (CD/NCD), or treatment group (no treatment, NSAID < 1 wk, NSAID > 1 wk, corticosteroids (cort) < 1 wk, cort > 1 wk, other). **c**. PGE_2_ levels expressed as pg/μg DNA were significantly lower in non-herniated samples compared with extruded and protruded samples in NCD dogs, regardless of the tissue origin (NP/AF), or treatment group (no treatment, NSAID < 1 wk, NSAID > 1 wk, corticosteroids (cort) < 1 wk, cort > 1 wk, other). ** Indicate significant difference at a 99 % confidence level

Pfirrmann grade II samples from this study were compared with Pfirrmann grade II samples obtained from experimental CD dogs (Fig. 3a) [40]. As sample weights were not available in the previous study, PGE_2_ was normalized for DNA. In this combined Pfirrmann grade II dataset, PGE_2_/DNA in the NP was significantly higher in extruded samples compared with Pfirrmann grade II IVDs with the NP in situ. To compare this combined Pfirrmann grade II dataset to the complete dataset, we have normalized PGE_2_ for DNA (Fig. 3b and c).

### Histology and COX-2 expression

Histological scores according to the grading scheme by Bergknut et al. ranged from 7 – 12 (median = 8) for Pfirrmann grade1, 8 – 27 (median = 14) for Pfirrmann grade II, 14 – 20 (median =19) for Pfirrmann grade III, and 18 – 26 (median = 21) for Pfirrmann grade IV + V. In 5/37 IVDs from 3/16 dogs ventral bone formation was seen in grade I – V IVDs. Histology revealed no inflammatory cells or fibroblasts in the NP of Pfirrmann grade I – V IVDs, and in the dorsal AF of Pfirrmann grade I IVDs. However, in higher degeneration grades, focal infiltration of macrophages, proliferation of fibroblasts and capillaries were detected in the dorsal and/or the ventral ligament, extending into the outer layers of the dorsal and ventral AF, respectively (Fig. 3). Macrophages and proliferation of fibroblasts were present in 0 % (0/10), 10 % (1/10), 83 % (5/6) and 55 % (6/11) of the IVDs scored a Pfirrmann grade I, II, III, IV + V, respectively. Numbers of macrophages and fibroblasts in grade IV + V IVDs were significantly higher than in grade I, and in grade III significantly higher than in grade I and II. Protrusion of the AF was seen in a grade II and a grade IV + V IVD.

**Fig. 4:**
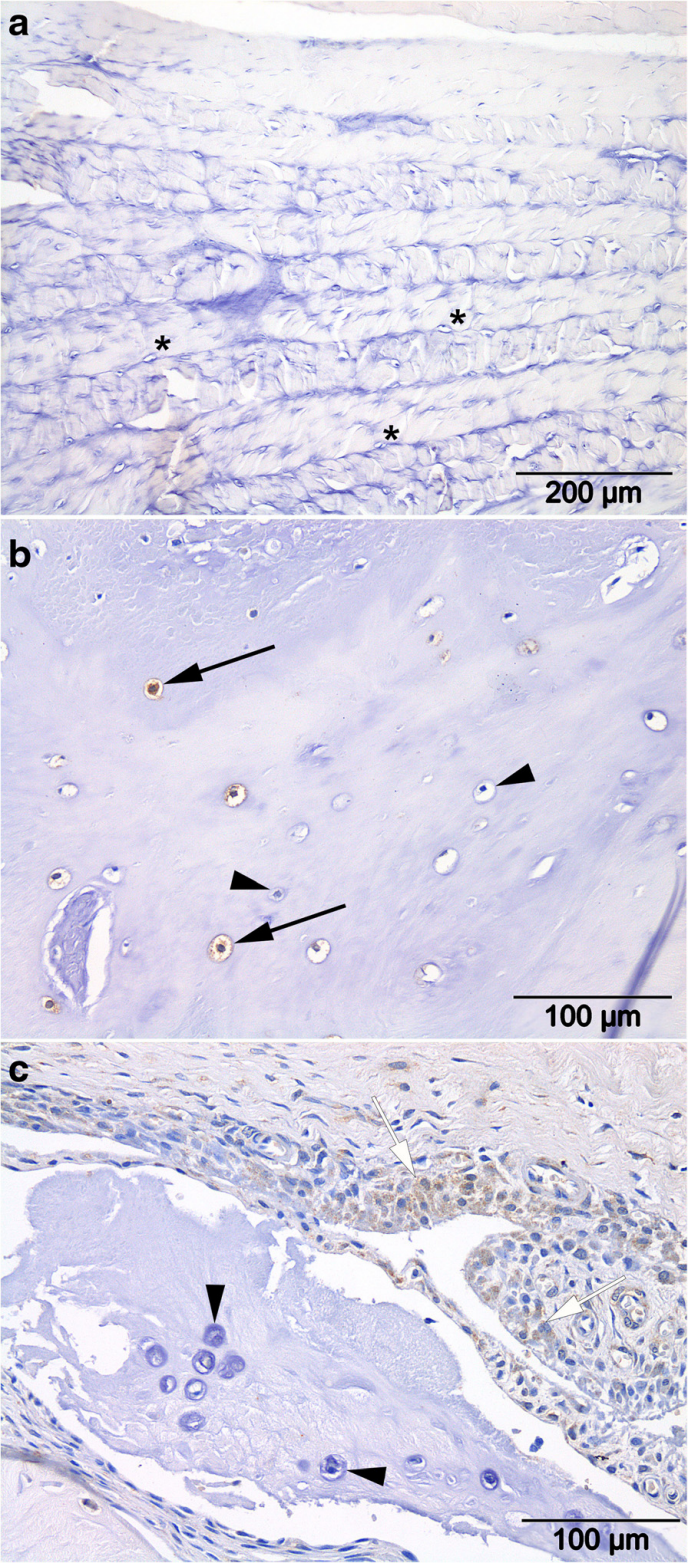
Representative histological images of the annulus fibrosus (AF) of intervertebral discs (IVDs) graded according to Pfirrmann stained with a COX-2 antibody and counterstained with hematoxylin. **a**. The dorsal AF of a non-degenerated Pfirrmann grade 1 IVD consisted of well-organized lamellae with COX-2 negative spindle-shaped fibroblasts (*asterisks*). **b**. In the dorsal AF of a degenerated Pfirrmann grade IV IVD lamellar organization was lost and COX-2 negative chondrocytes (*arrowheads*) as well as COX-2 positive chondrocytes (*arrows*) were present. **c**. The dorsal AF of a Pfirrmann grade V IVD consisted of COX-2 negative chondrocytes (*arrowheads*), whereas COX-2 positive macrophages (*open arrows*) were situated in the dorsal ligament

**Fig. 5:**
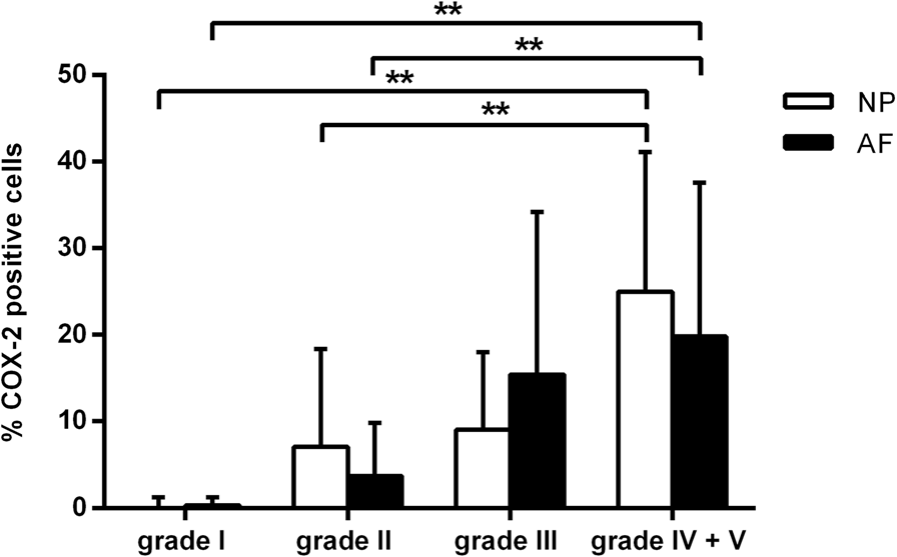
Percentage of COX-2-positive cells in the nucleus pulposus (NP) and annulus fibrosus (AF) per Pfirrmann grade. The NP and AF of grade I and grade II samples were significantly lower compared with the NP and AF grade IV + V samples. ** Indicate significant difference at a 99 % confidence level

Percentages of COX-2-positive cells in the NP and dorsal AF of grade I and grade II tissue were significantly lower compared with the NP and dorsal AF of grade IV + V samples (Fig. 4 and Fig. 5). The presence of macrophages and fibroblasts in the dorsal AF was moderately correlated (Spearman’s ρ = 0.4, *p-*value = 0.003) with COX-2 positive cells in the dorsal AF.

## Discussion

To our knowledge this is the first study that describes levels of COX-2, PGE_2_, cytokines, chemokines, and matrix components in IVDs from CD and NCD dogs with and without clinical signs of IVD disease and degeneration. PGE_2_ levels were significantly higher in degenerated IVDs compared with non-degenerated IVDs, and they were also higher in herniated (protruded and extruded) IVDs from NCD dogs compared with non-herniated IVDs of NCD dogs. In contrast to PGE_2_ levels in Pfirrmann grade II IVDs from CD dogs with (a limited number of) protruded IVDs, PGE_2_ levels in extruded IVDs were not significantly different from IVDs with the NP in situ. Furthermore, COX-2 protein expression was significantly higher in degenerated IVDs compared with non-degenerated IVDs. These results are consistent with findings in herniated human lumbar IVD cells that produced increased PGE_2_ levels spontaneously in vitro compared with PGE_2_ levels in control IVD cells [15].

Contrary to PGE_2_ levels, COX-2 expression in the NP and AF and numbers of macrophages in the dorsal and ventral ligaments were increased in advanced stages of degeneration. Histological results of non-herniated degenerated IVDs in our study are consistent with histological findings described in studies on canine herniated IVDs. In extruded IVDs an acute inflammatory reaction has been described, characterized by neutrophils and macrophages, while in protruded IVDs a more chronic inflammatory reaction has been described, characterized by macrophages, lymphocytes and plasma cells [46, 47]. Macrophages do not only have a phagocytic function, but secret next to cytokines also a number of growth factors, e.g. fibroblast growth factor, transforming growth factor beta, that can induce neovascularization and mediate cell proliferation and differentiation [48]. The focal infiltrates of macrophages, proliferation of fibroblasts and capillaries, and the new bone formation as was seen histologically in some IVDs are reactive tissue changes that might reflect a process of tissue repair. Although a physiological inflammatory response to aseptic tissue injury primarily serves to promote tissue repair, macroscopic findings may reflect an excessive inflammatory response. This response may have detrimental effects on tissue integrity, and may contribute to the pathogenesis of IVD degeneration and/or disease.

Significantly higher PGE_2_ levels in degenerated NP tissues compared with non-degenerated IVDs were observed. Although not significantly different, PGE_2_ levels and COX-2 expression in grade II and IV + V IVDs were consistently higher in the NP of degenerated IVDs compared with AF, while the contrary was true for non-degenerated IVDs. In Pfirrmann grade II samples from CD dogs, PGE_2_ levels in the NP were significantly higher in Pfirrmann grade II IVDs with protrusion compared with IVDs with the NP in situ. This may indicate that the production of inflammatory mediators is more pronounced at the NP level. We cannot exclude that NP and AF cells respond differently to inflammatory stimuli and mechanic stress and hence produce different levels of PGE_2_, as also suggested by others based on in vitro experiments in rat IVD cells [49]. Cytokine and chemokine profiles in this study are largely consistent with limited veterinary publications. The significantly increased CCL2 levels in NP tissue of dogs with Hansen type I herniation compared with AF tissue and NP tissue in dogs with Hansen type II herniation are in line with other studies reporting upregulated gene expression levels of CCL2 in dogs with extrusion of the NP [35]. Furthermore, increased CCL2 protein expression and CCL2 production levels have been reported in human prolapsed IVDs [28]. No studies in canine tissues, and only limited studies in human tissues, have determined cytokine and/or chemokine levels by using a (multiplex) sandwich immunoassay, and have shown increased levels of IFN-γ, IL-1, TNF-α, and CCL2, in epidural lavage fluid and in cell culture media, which complicates comparison of results [51–53]. Cytokine levels of IL-2, IL-6, IL-7, IL-8, IL-10, IL-15, IL-18, IP-10, TNF-α, and GM-CSF were not detected in NP and AF tissues of this study, consistent with downregulated gene expression levels of IL-2, IL-6, IL-10, and TNF-α in herniated canine IVDs [35]. Nevertheless, our findings seem to be in contrast with increased protein and gene expression levels of TNF-α and IL-1 in human IVDs [23–25, 28, 29, 54]. Given the short half-life of TNF- α and cytokines [55, 56], and that all samples collected from degenerated IVDs were obtained during surgery and in several cases after flushing of the spinal canal, we cannot rule out degradation of cytokines/chemokines by the collection, preparation, and storage process. Although several studies have shown that IVD cells have the capacity to produce PGE_2_, we cannot rule out that PGE_2_ levels were influenced by infiltration from the epidural space.

Despite elevated PGE_2_ levels in degenerated NP tissue in this study, GAG content was not significantly different between healthy and degenerated IVDs, while severely degenerated AFs (grade IV + V) had a higher GAG content compared with the NP. The latter may be explained by the presence of GAG-producing chondrocytes in the AF, known to be present in later stages of degeneration [45], or by the presence of unidentified GAG-rich herniated NP and/or inner AF material in AF samples. These findings are in contrast with the decrease in GAG content with increasing IVD degeneration described in literature [1, 11]. One plausible explanation for this discrepancy lies in the scoring system of degeneration prior to surgery and the matrix heterogeneity of the degenerated NP tissue, discussed in detail below. Interestingly, cell density (DNA/weight) in our study was significantly higher in the NP of severely degenerated IVDs compared with mildly degenerated IVDs. These findings touch upon findings in human IVD degeneration, in which cell density in the inner AF and NP of severely degenerated (Thompson grade V) specimens was significantly higher compared with lower grades [57, 58].

The results on the effects of PGE_2_ on proteoglycan metabolism are conflicting.PGE_2_ at concentrations much lower than those involved in inflammation have been demonstrated to be chondroprotective [59]. PGE_2_ has been described to have anti-catabolic effects by downregulating the expression and synthesis of IL-1, TNF- α, and matrix metalloproteinases (MMPs), and to have anabolic effects by to inducing the expression, synthesis and secretion of IGF-I, and stimulating collagen and proteoglycan synthesis, important factors in anabolic processes [16, 60–62]. In vitro, low concentrations of PGE_2_ have been described to stimulate proteoglycan synthesis in rat chondrocytes, whereas higher doses have been described to decrease proteoglycan synthesis in NP cells [16, 61]. Furthermore, degradation of proteoglycans was not inhibited by a range of PGE_2_ concentrations in osteoarthritic chondrocytes [60]. These possible protective effects of PGE_2_ might have resulted in preservation of GAG content in the course of IVD degeneration. Nevertheless, these results should be interpreted with care, as GAG content of the studied tissues may have been affected by confounding factors explained below.

There are several confounding factors that may affect the results in the current study, including the factors that influence the scoring system of degeneration and the matrix heterogeneity of the degenerated NP tissue. Extruded NP tissue displaced into the vertebral canal results in narrowing of the disc space and a T2-hypointense area within the IVD on MRI. Hence, we cannot exclude that prior to the extrusion incident the IVD may have been assessed with a lower Pfirrmann score. Moreover, in CD dogs, calcification of the NP could have negatively influenced the signal intensity in the NP [63, 64]. In addition, in both CD and NCD dogs, IVDs may have been graded falsely higher due to hemorrhage or inflammation, that may have influenced the appearance of the IVD on MR images. Matrix heterogeneity is common in degenerating NP tissue. In human IVDs several disc-specific locations are described with a high variation in GAG and water content, suggestive of focal damage and degeneration [65]. Although this has not yet been described in dogs, we cannot rule out that tissues collected during surgery may have originated from specific GAG-rich areas in the IVD, that inherently are more prone to extrusion/protrusion compared with degenerated fibrotic tissue. Furthermore, due to sample limitations PGE_2_ values higher than 1000 pg/ml could not be measured reliably, but could have resulted in an underestimation of the highest samples. Lastly, a relatively high percentage of dogs in this study was treated prior to surgery with anti-inflammatory drugs, e.g. NSAIDs and corticosteroids. Dogs that did not respond to anti-inflammatory drugs initially, were treated with other drugs, e.g. opioids, GABA-agonists. Although treatment groups were categorized, duration of treatment and dosages used showed a high variation, and might have had an influence on the results.

From a clinical perspective, decompression surgery is recommended if dogs present with clinical signs, and diagnostic work-up indicates compression of neural tissue (spinal cord and/or nerve roots) due to extruded material. With regard to an intradiscal application that provides controlled release of an anti-inflammatory drug, future studies should focus on protruded IVDs. Obviously, this would indicate development of an application in NCD dogs, as disc protrusion rarely occurs in CD dogs. IVDs ideally should be early degenerated (Pfirrmann grade II – III), without irreversible anatomical malformations due to degenerative changes.

## Conclusion

In this study we have shown that PGE_2_ levels, and CCL2 levels in degenerated and herniated tissues were significantly higher compared with non-degenerated and non-herniated tissues. COX-2 expression in the NP and AF and numbers of macrophages in the AF increased with advancing degeneration stages. Although macrophages invade the dorsal and ventral AF as degeneration progresses, the production of inflammatory mediators seems most pronounced in degenerated NP tissue. Future studies are needed to investigate if inhibition of PGE_2_ levels in degenerated IVDs provide effective analgesia and exerts a protective role in the process of IVD degeneration and the development of IVD disease.

## Additional files

Additional file 1:
**A more detailed representation of included samples in addition to Table** 1 **in the original article.** (DOCX 29 kb) Additional file 2:
**Significant differences and confidence intervals of statistical analyses.** (DOCX 25 kb)

## Abbreviations

AF: Annulus fibrosus
AIC: Akaike information criterion
BSA: bovine serum albumin
CCL2: chemokine (C-C motif) ligand 2
CD: chondrodystrophic
Cort: corticosteroids
COX: cyclooxygenase
CT: computed tomography
CXCL1: chemokine (C-X-C motif) ligand 1
DEC: Animal Experiments Committee (Dutch: Dierexperimentencommissie)
DMMB: dimethylmethylene blue
EDTA: ethylenediaminetetraacetic acid
GAG: glycosaminoglycan
GM-CSF: granulocyte-macrophage colony-stimulating factor
IL: interleukin
IVD: intervertebral disc
MMP: matrix metalloproteinases
MR: magnetic resonance
NCD: non-chondrodystrophic
NP: nucleus pulposus
NSAIDs: non-steroidal anti-inflammatory drugs
PGE_2_: Prostaglandin E_2_
TBS: tris buffered saline
TNF-α: tumor necrosis factor α
T2W: T2-weighted
