# Supplementary material for: Inflammatory profiles in canine intervertebral disc degeneration
Source: BMC Vet Res. 2016 Jan 13;12:10. doi: 10.1186/s12917-016-0635-6 (PMC4711078; doi:10.1186/s12917-016-0635-6)
Supplement: Additional file 1: — A more detailed representation of included samples in addition to Table 1 in the original article. (DOCX 29 kb) [file 12917_2016_635_MOESM1_ESM.docx]

**Additional file 1.**  A more detailed representation of included samples in addition to Table 1 in the original article.

|  | **NP-CD** | **NP in situ/**  **Extr/Protr** | **Treatment** | **NP-NCD** | **NP in situ/**  **Extr/Protr** | **Treatment** | **AF-CD** | **NP in situ/**  **Extr/Protr** | **Treatment** | **AF-NCD** | **NP in situ/**  **Extr/Protr** | **Treatment** | |
| --- | --- | --- | --- | --- | --- | --- | --- | --- | --- | --- | --- | --- | --- |
| **Grade I** | **0** | **0** |  | **9** | **9** | 9: No treatment | **0** | **0** |  | **10** | **10** | 10: No treatment | |
|  |  | **0** |  |  | **0** |  |  | **0** |  |  | **0** |  |  |
|  |  | **0** |  |  | **0** |  |  | **0** |  |  | **0** |  |  |
| **Grade II** | **15** | **8** | 7: No treatment | **9** | **0** |  | **15** | **6** | 6: No treatment | **9** | **0** |  |  |
|  |  |  | 1: NSAID > 1 wk |  | **3** | 1: NSAID < 1 wk |  | **6** | 3: NSAID < 1 wk |  | **2** | 1: NSAID < 1 wk | |
|  |  | **6** | 2: NSAID < 1 wk |  |  | 2: NSAID > 1 wk |  |  | 1: NSAID > 1 wk |  |  | 1: NSAID > 1 wk | |
|  |  |  | 2: NSAID > 1 wk |  | **6** | 4: No treatment |  |  | 1: Cort < 1 wk |  | **7** | 4: No treatment | |
|  |  |  | 1: Cort < 1 wk |  |  | 1: NSAID > 1 wk |  |  | 1: Cort > 1 wk |  |  | 2: NSAID > 1 wk | |
|  |  |  | 1: Cort > 1 wk |  |  | 1: Cort > 1 wk |  | **3** | 1: No treatment |  |  | 1: Cort > 1 wk | |
|  |  | **1** | 1: Cort > 1 wk |  |  |  |  |  | 1: NSAID > 1 wk |  |  |  |  |
| **Grade III** | **7** | **0** |  | **8** | **0** |  | **6** | **0** |  | **8** | **0** |  |  |
|  |  | **6** | 2: NSAID < 1 wk |  | **2** | 1: NSAID < 1 wk |  | **5** | 3: NSAID < 1 wk |  | **1** | 1: Other |  |
|  |  |  | 1: Cort < 1 wk |  |  | 1: Cort < 1 wk |  |  | 2: NSAID > 1 wk |  | **7** | 2: No treatment | |
|  |  |  | 1: Cort > 1 wk |  | **6** | 1: No treatment |  | **1** | 1: Cort > 1 wk |  |  | 1: NSAID < 1 wk | |
|  |  |  | 1: Other |  |  | 2: NSAID < 1 wk |  |  |  |  |  | 2: NSAID > 1 wk | |
|  |  |  | 1: NA |  |  | 2: NSAID > 1 wk |  |  |  |  |  | 2: Other |  |
|  |  | **1** | 1: Cort > 1 wk |  |  | 1: Cort < 1 wk |  |  |  |  |  |  |  |
| **Grade** | **13** | **1** | 1: NSAID < 1 wk | **10** | **0** |  | **9** | **1** | 1: Cort < 1 wk | **14** | **0** |  |  |
| **IV+V** |  | **12** | 2: NSAID < 1 wk |  | **7** | 3: NSAID < 1 wk |  | **8** | 2: No treatment |  | **4** | 1: NSAID < 1 wk | |
|  |  |  | 4: NSAID > 1 wk |  |  | 3: NSAID > 1 wk |  |  | 2: NSAID < 1 wk |  |  | 1: NSAID > 1 wk | |
|  |  |  | 2: Cort < 1 wk |  |  | 1: Cort > 1 wk |  |  | 2: NSAID > 1 wk |  |  | 1: Cort > 1 wk | |
|  |  |  | 1: Cort > 1 wk |  | **3** | 1: Other |  |  | 1: Cort > 1 wk |  |  | 1: Other |  |
|  |  |  | 2: Other |  |  | 2: NA |  |  | 1: Other |  | **10** | 4: No treatment |  |
|  |  |  | 1: NA |  |  |  |  | **0** |  |  |  | 1: NSAID < 1 wk | |
|  |  | **0** |  |  |  |  |  |  |  |  |  | 3: NSAID > 1 wk | |
|  |  |  |  |  |  |  |  |  |  |  |  | 1: Other |  |
|  |  |  |  |  |  |  |  |  |  |  |  | 1: NA |  |
|  | **Total** |  |  | **Total** |  |  | **Total** |  |  | **Total** |  |  |  |
|  | **35** | **9** |  | **36** | **9** |  | **30** | **7** |  | **41** | **10** |  |  |
|  |  | **24** |  |  | **12** |  |  | **19** |  |  | **7** |  |  |
|  |  | **2** |  |  | **15** |  |  | **4** |  |  | **24** |  |  |

**NP = nucleus pulposus, CD = chondrodystrophic, Extr = extrusion, Protr = protrusion, NCD = non-chondrodystrophic,AF = annulus fibrosus,**

**NSAID = non-steroidal anti-inflammatory drug, Cort = corticosteroids, NA = not available**
