# Supplementary material for: Inflammatory profiles in canine intervertebral disc degeneration
Source: BMC Vet Res. 2016 Jan 13;12:10. doi: 10.1186/s12917-016-0635-6 (PMC4711078; doi:10.1186/s12917-016-0635-6)
Supplement: Additional file 2: — Significant differences and confidence intervals of statistical analyses. (DOCX 25 kb) [file 12917_2016_635_MOESM2_ESM.docx]

**Additional file 2. Significant differences and confidence intervals of statistical analyses**

Tables 1, 2, and 3 represent significant differences and confidence intervals of statistical analyses. Figure numbers correspond to figures shown in the main article. Table 4 represents the significant differences and confidence intervals of statistical analysis of data represented in additional file 2.

**Table 1.** Significant differences and confidence intervals of statistical analyses of GAG and DNA data.

| ***Figure 1.*** Mean +standard deviation GAG and DNA content normalized for weight in the nucleus pulposus (NP) and annulus fibrosus (AF) per Pfirrmann grade (A, B) and per herniation (C, D). | | | | | | |
| --- | --- | --- | --- | --- | --- | --- |
| ***Degeneration*** | | | | | | |
| ***1A. GAG/weight*** |  | | **Estimated coefficient** | **Confidence interval (CI)** | **CI (%)** | |
| NP grade 4+5 | **vs** | AF grade 4+5 | 0.65 | 0.05 – 1.25 | 99 | |
| ***1B. DNA/weight*** |  | | **Estimated coefficient** | **Confidence interval (CI)** | **CI (%)** | |
| NP grade 2 | **vs** | NP grade 4+5 | 1.13 | 0.22 – 2.04 | 99 | |
|  |  |  |  |  |  |  |

*GAG = glycosaminoglycan, DNA = deoxyribonucleic acid*

**Table 2**. Significant differences and confidence intervals of statistical analyses of PGE_2_ and cytokine data.

| ***Figure 2.*** Mean + standard deviation PGE_2_ and chemokine (CCL2 and CXCL1) levels normalized for weight and DNA in the nucleus pulposus (NP) and annulus fibrosus (AF) per Pfirrmann grade (**A, B, C, D**) and per herniation (**E, F, G, H**). | | | | | | |
| --- | --- | --- | --- | --- | --- | --- |
| ***Degeneration*** | | | | | | |
| ***2A. PGE_2_/weight*** |  |  | **Estimated coefficient** | **Confidence interval (CI)** | **CI (%)** | |
| NP grade 2 | **vs** | NP grade 1 | 2.54 | 1.10 – 3.99 | 99 | |
| NP grade 3 |  | NP grade 1 | 2.84 | 1.28 – 4.41 | 99 | |
| NP grade 4 |  | NP grade 1 | 2.67 | 1.20 – 4.14 | 99 | |
| ***2B. PGE_2_/DNA*** |  |  | **Estimated coefficient** | **Confidence interval (CI)** | **CI (%)** | |
| Grade 2 | **vs** | Grade 1 | 2.17 | 1.18 – 3.17 | 99 | |
| Grade 3 |  | Grade 1 | 2.13 | 1.04 – 3.21 | 99 | |
| Grade 4 |  | Grade 1 | 1.47 | 0.46 – 2.48 | 99 | |
| ***Herniation*** | | | | | | |
| ***2E. PGE_2_/weight*** |  |  | **Estimated coefficient** | **Confidence interval (CI)** | **CI (%)** | |
| Extrusion NCD | **vs** | In situ NCD | 1.85 | 0.31 – 3.40 | 99 | |
| Protrusion NCD |  | In situ NCD | 1.98 | 0.79 – 3.17 | 99 | |
| ***2F. PGE_2_/DNA*** |  |  |  |  |  | |
| Extrusion NCD | **vs** | In situ NCD | 1.85 | 0.31 – 3.40 | 99 | |
| Protrusion NCD |  | In situ NCD | 1.98 | 0.79 – 3.17 | 99 | |
| ***2H. CCL2/weight*** |  |  | **Estimated coefficient** | **Confidence interval (CI)** | **CI (%)** | |
| NP extrusion | **vs** | AF extrusion | 2.07 | 0.64 – 3.51 | 99 | |
| NP extrusion |  | NP protrusion | 1.33 | 0.21 – 2.45 | 99 | |
|  |  |  |  |  |  |  |

*PGE_2_ = prostaglandin E_2_, CCL2 = chemokine (C-C motif) ligand 2, NCD = non-chondrodystrophic*

| **Table 3.** Significant differences and confidence intervals of statistical analyses performed on COX-2 expression data, corresponding to Figure 3 shown in the main article. | | | | | |
| --- | --- | --- | --- | --- | --- |
| ***Figure 3. Percentage of COX-2-positive cells in the nucleus pulposus (NP) and annulus fibrosus (AF) per Pfirrmann grade.*** | | | | | |
|  |  |  | **Estimate hazard ratio (HR)** | **Confidence interval (CI)** | **CI (%)** |
| NP grade 4+5 | **vs** | NP grade 1 | 34.3 | 2.09 – 562.54 | 99 |
| NP grade 4+5 |  | NP grade 2 | 7.40 | 1.39 – 39.46 | 99 |
| AF grade 4+5 |  | AF grade 1 | 27.32 | 1.78 – 419.75 | 99 |
| AF grade 4+5 |  | AF grade 1 | 4.92 | 1.64 – 14.79 | 99 |

*COX-2 = cyclooxygenase 2*

**Table 4.** Significant differences and confidence intervals of statistical analyses of PGE_2_ and DNA data for grade 2 samples only, corresponding to additional file 2.

| ***Additional file 2. Percentage of COX-2-positive cells in the nucleus pulposus (NP) and annulus fibrosus (AF) per Pfirrmann grade. The NP and AF of grade 1 and grade 2 samples were significantly lower compared with the NP and AF grade 4 + 5 samples.*** | | | | | |
| --- | --- | --- | --- | --- | --- |
|  |  |  | **Estimated coefficient** | **Confidence interval (CI)** | **CI (%)** |
| NP protrusion | **vs** | NP in situ | 2.08 | 1.06 – 3.10 | 99 |
